# Case of Copper-Coloured Syphilitic Eruption Affecting the Conjunctiva

**Published:** 1845-03

**Authors:** Alfred Smee


*Case of Copper-Coloured Syphilitic Eruption affecting the Conjunctiva.* By Alfred Smee, F. R. S.—As far as I know, there is no record of specific eruptions attacking the conjunctiva, but an instance having recently occurred at the Central London Ophthalmic Hospital, it becomes my duty to record it.

The subject of the affection was a respectable married female, the wife of a traveller, who applied at the Charity to be relieved of a small ulcer which existed at the edge of the eyelid. From its appearance I immediately recognised its specific character, and upon inquiry, I learnt that both husband and wife had laboured under syphilis for a period of two or three years. At that time I put her upon the general antimonial treatment, described in the Medical Gazette, and the ulceration speedily healed. At this period there were also numerous copper-coloured spots over the skin, which I supposed she thought of but little consequence, as I lost sight of her as soon as the ulceration had healed.

After a short interval she returned again, and stated that her eye was somewhat uneasy, and that she feared a return of the malady: on examination the conjunctiva below the cornea presented a spot a little smaller than a silver penny-piece. This spot appeared formed by the conjunctiva itself at that point being swollen; in fact, the surface was obviously raised, and the colour changed to that of a cupreous hue, somewhat similar to the colour of the spots of the skin, but considerably lighter: this spot was not absolutely opaque, but semi-transparent, and it gave the idea that the conjunctiva at that part was swollen in its substance, and tinged of a copper colour. It presented no unusual vascularity; in fact, I do not know that I should be justified in asserting that there was a single vessel either increased in size or added in number to those existing in the normal state. The copper colour, then, had no immediate reference to the vessels, and was due to the part exhibiting the syphilitic stain; a phenomenon by no means understood.

This eruption must be considered analogous to those of measles, small-pox, and scarlet fever, which commonly attack that membrane; but perhaps it is even more analogous to that of purpura, which often affects the conjunctiva, of which a very beautiful example occurred at this Charity last summer. The spots of the two diseases, although presenting the distinct characters appertaining to each malady, were perfectly analogous; that of one exhibiting deep purple circumscribed spots, that of the other a copper-coloured, circumscribed spot.

I remember to have seen some time since pustules on the conjunctiva, associated with pustules on the skin, which appeared to have their origin in the syphilitic virus, but up to the present time I have never before seen the copper-coloured eruption to affect this membrane.

There is no particular practical importance to be attached to this malady, for it causes very little trouble of itself, and requires no treatment apart from that required by the system generally. In this case the antimonial treatment was employed, but as the progress was not to my mind sufficiently rapid, I tried the iodide of potassium, giving four grains three times a day, under which treatment it declined, and the patient discontinued her attendance.

The occurrence of this eruption is to me exceedingly interesting, inasmuch as, being the first case of the kind which has been narrated, it opens the door for further investigation. The affection, in this instance, occurred in a case where the poison had run its own course for years, without regular treatment, and therefore the rarity of the affection may be increased by this circumstance of itself being unusual. My motive in recording this case is to direct the profession to its occurrence, in the hope that by a multitude of observations we may ascertain to what extent this disease attacks the conjunctiva.—*Lond. Med. Gaz.*

